# A Tree Attenuation Factor Model for a Low-Power Wide-Area Network in a Ruby Mango Plantation †

**DOI:** 10.3390/s24030750

**Published:** 2024-01-24

**Authors:** Supachai Phaiboon, Pisit Phokharatkul

**Affiliations:** 1 Department of Electrical Engineering, Faculty of Engineering, Mahidol University, 999 Salaya, Nakhorn Pathom 73170, Thailand; 2 Department of Electrical Engineering and Energy Management, Faculty of Engineering, Kasem Bundit University, Suanluang, Bangkok 10250, Thailand; pisit.pho@kbu.ac.th

**Keywords:** RSSI, Ruby mango, LPWAN 433 MHz, smart agriculture, path loss prediction

## Abstract

Ruby mangoes are a cultivar with a thick skin, firm texture, red color, no splinters, and thin seeds that is grown in eastern Thailand for export. Implementing a low-power wide-area network (LPWAN) for smart agriculture applications can help increase the crop quality or yield. In this study, empirical path loss models were developed to help plan a LPWAN, operating at 433 MHz, of a Ruby mango plantation in Sakaeo, eastern Thailand. The proposed models take advantage of the symmetric pattern of Ruby mango trees cultivated in the plantation by using tree attenuation factors (TAFs) to consider the path loss at the trunk and canopy levels. A field experiment was performed to collect received signal strength indicator (RSSI) measurements and compare the performance of the proposed models with those of conventional models. The proposed models demonstrated a high prediction accuracy for both line-of-sight and non-line-of-sight routes and performed better than the other models.

## 1. Introduction

Thailand is famous for its mangoes; in particular, Ruby mangoes are a cultivar with a thick skin, firm texture, red color, no splinters, and thin seeds that is grown in eastern Thailand for export. The Thailand Board of Investment supported 71 projects in the agriculture and food processing sector worth 375.31 million USD in 2019 and 92 projects worth 882.12 million USD in 2022 for a 30% increase in investment each year [1]. Smart agriculture is widely used in many countries to accommodate extreme weather conditions, growing populations, and a reduction in agriculture areas. It involves the use of different types of Internet of Things (IoT) sensors to collect data on the soil, air, water, and insects, which are then analyzed to facilitate the decision-making process [2]. Using IoT to monitor mango plantations can help control the quality and quantity of the crop as well as increase the operational efficiency and crop productivity [3]. Jani and Chaubey [4] studied the automation of watering, fertilizing, pest detection, and pesticide spraying to minimize farmer intervention.

Long Range (LoRa) low-power wide-area networks (LPWANs) are the preferred communications option of most IoT applications for smart agriculture. LoRa-based solutions have been applied to monitor irrigation systems, crops, trees, and livestock, but potential issues include the network bandwidth, density, sensor complexity, and power demand, as well as latency in the decision-making process [5]. LoRa uses license-free and region-dependent industrial, scientific, and medical (ISM) frequency bands: 863–870 MHz for Europe and 902–928 MHz for the United States. It can also be set to operate in the lower ISM bands of 433 and 169 MHz [6]. Pinto et al. [7] used an ISM band of 2.4 GHz in a tomato greenhouse. Abouzar et al. [8] studied the received signal strength indicator (RSSI) of a wireless sensor network (WSN) operating at 2.45 MHz above and below the canopy in an agricultural field. Increasing the power efficiency and network life are important issues for WSNs. The key factors are the data packet size and transmission power level [9]. Plantations comprise rows of densely foliated trees, which can cause a significant propagation loss. Moreover, tree leaves tend to absorb water, which can cause further scattering of the signal. Low frequencies, such as 240 MHz, are less likely to be affected by weather conditions, such as rain and strong winds [10]. Identifying the communication channel pattern is important for describing the occurrence and nature of large-scale fading effects [11]. LoRa data transmission is vulnerable to near-ground effects and blockages caused by vegetation canopies and tree trunks, which interrupt the communication nodes and increase their energy consumption.

### 1.1. Related Work

There are generally two types of path loss models. Firstly, the most commonly used are empirical path loss models for vegetation, namely, the log-distance model and exponential decay model (EDM), which are derived from the measured data in real environments, therefore are easy to use and provide an accuracy prediction. The second type are machine learning models, which are derived from training processes of expert systems.

In case of the empirical models, Raheemah et al. proposed an empirical path loss model for mango greenhouses, at a frequency of 2.425 GHz, with seven different antenna heights of 0.5 m, 1.0 m, 1.5 m, 2 m, 2.5 m, 3 m, and 3.5 m [12]. In this study, thirteen mango trees were in a row, with a total of 3 rows. Also, the separation distance between each tree in the same row was approximately 3.2 m, and the separation distance between each row was 2.2 m. The trees were 5 years old with a mean maximum height of 2 m, a main trunk height of 1 m, and a mean trunk diameter of 0.16 m. This model showed the best prediction compared with the conventional models. Anzum et al. proposed a log-distance with multi-wall attenuation model, based on LoRa, at 433 MHz measured data for a symmetric pattern of oil palm trees in plantation [13]. This model provided an average RMSE of 2.74 with respect to the measured path loss. Anderson et al. proposed characterization of low-antenna (1.3 m) with an ultrawideband pulse (830–4200 MHz), for four forest environments, light brush, light forest, medium forest, and dense forest. The results showed that the path loss exponent (*n*) ranged from 2.5 to 3.8 with a standard deviation (*σ*) range from 2.1 dB to 4.4 dB [14]. Azevedo et al. proposed an empirical path loss model via the tree trunks of different trees at frequencies of 870 MHz and 2.414 MHz. The multiplication of the tree density by the average diameter of the trunk was a parameter that influenced the path loss exponent [15]. Additionally, tree density, average tree canopy diameter, and foliage density were input parameters to estimate the path loss in areas with tree foliage [16]. Barrios-Ulloa et al. reviewed the path loss models for 200 MHz to 95 GHz, in both the log-distance and ABC models and provided a comparison of the RMSE of those models [17]. A wireless communication near-ground was proposed with the plane earth model for VHF and UHF bands by Meng et al. The researchers limited their interest to specific phenomena, such as the impact of near-ground or surface components on signal propagation in different environments [18]. Additionally, path loss models with break point distance on-ground, near-ground, and above-ground (5 cm, 50 cm and 1 m) were measured and analyzed at a frequency of 470 MHz by Tang et al. [19]. Jong et al. proposed a tree scattering model for a single oak tree at a frequency of 1.9 GHz [20]. Lastly, Leonor et al. proposed a raytracing-based scattering model for a Ficus benjamina tree and a Thuja pelicata tree at frequencies of 20 GHz and 62.4 GHz [21,22].

There are many types of machine learning (ML) models, such as the ANFIS model by Hakim et al. [23], Artificial Neural Network (ANN) by Wu et al. [24] and Pedro et al. [25], and Asynchronous Federated ML by You et al. [26]. Additionally, Pal et al. proposed non-dominated sorting genetic algorithm (NSGA-III) models for two medium grass vegetations, paddy and sugarcane, using 2.4 MHz measured data over node height and crop cycle periodic combinations [27]. These models provided high performance for each trained point, which were confirmed by Shibu et al. [28]. However, when the environments are changed, the models may require new training or optimization to avoid predicted errors. 

The above empirical path loss models depended on vegetation types, the radio wave frequency, vegetation height, and the distance in the depth of the vegetation. However, all of them provided an estimate of the path loss of the radio signal that is quite different from those obtained from the conventional standard models. Therefore, in this study, propagation models at 433 MHz for the WSN at trunk and canopy levels with tree attenuation factors (TAFs) are proposed for a Ruby mango plantation, with the addition of LOS path loss models. Additionally, the excess free-space loss or ABC models are included and compared. Furthermore, we applied the near-ground plane earth model to optimize the accuracy of the proposed model. Finally, a comparison of the proposed models and standard models such as, ITU, COST, and FITU are presented and discussed.

### 1.2. Contribution

A common source of error in path loss models is non-uniform vegetation. However, Ruby mango trees are trimmed to limit their height and are planted in symmetric patterns. Therefore, the size of the trees remains much the same despite changes in the trees over time. In this study, this fact was used to propose empirical path loss models for planning the WSN of a Ruby mango plantation in Sakaeo, eastern Thailand. For the proposed models, TAFs were used to consider non-line-of-sight (NLOS) path losses at the trunk and canopy levels, which were then combined with line-of-sight (LOS) path losses. The proposed TAFs are non-linear for each TAF and are a characteristic of this type of plantation. This contribution is valid because tree height and spacing are maintained over the years.

Machine learning models may provide better results, however, they require considerable time for computation. The proposed model is comfortable and uses little time for the computation. This study makes the following contributions to the literature:

- TAFs are proposed for a Ruby mango plantation. These factors can be used for both short- and long-distance path loss prediction with an accuracy comparable to conventional regression models.
- An exponential decay model is modified to be suitable for Ruby mango plantations.
- RSSI measurement data were captured for a LoRa LPWAN in the 433 MHz frequency channel.

The remainder of this paper is structured as follows: Section 2 presents the proposed empirical path loss models; Section 3 presents the field measurements; Section 4 presents the results and discussion; Section 5 concludes the paper.

## 2. Proposed Path Loss Models

The two main forms of empirical path loss models used for vegetation, the exponential decay (i.e., ABC) model and TAF model, were adopted in this study.

### 2.1. ABC Model

The following path loss model for a theoretically free-space can be used as a reference for estimating the path loss in different environments:

(1) $${PL}_{free}\;\left(dB\right)=-27.56+20{log}_{10}\left(f\right)+20{log}_{10}\left(d\right)\;\;\;\;\;$$

where *f* is the frequency (MHz) and *d* is the distance between transmitting and receiving antennas (m). For wave propagation through trees, the excess loss is generally expressed as:

(2) $${PL}_{excess}\;\left(dB\right)=Af^{B}d^{C}$$

where *A*, *B*, and *C* are values fitted to the measured data. For the near-ground path loss, a plane-Earth model is often used that considers direct rays (i.e., LOS), in addition to ground-reflected rays that are received by the receiver [18]:

(3) $${PL}_{Plane\;Earth}\left(dB\right)=40\log\left(d\right)-20\log\left(h_{t}\right)-20\log(h_{r})$$

where *h_t_* is the transmitting antenna height (m), *h_r_* is the receiving antenna height (m), and *d* is the distance between the transmitter and receiver (m). This model assumes that *d* is much greater than the *h_t_* and *h_r_*. The excess loss determined in (2) and the plane-Earth loss calculated in (3) can be combined to derive a path loss model for a forest:

(4) $${PL}_{forest}\left(dB\right)=Af^{B}d^{C}+40\log_{10}\left(d\right)-20\log_{10}\left(h_{t}\right)-20{log}_{10}(h_{r})$$

The effectiveness of the perfect plane-Earth model is reduced by lateral wave propagation from diffraction over treetops and around trees, especially in the VHF band. Thus, the fitted ground reflection model is often applied:

(5) $$L_{FGR}(dB)=10n{log}_{10}\left(d\right)-20{log}_{10}(h_{t})-20{log}_{10}\;(h_{r})$$

where *n* is an empirical path loss exponent. Then, the foliage path loss model is derived:

(6) $${PL}_{forest}\left(dB\right)=Af^{B}d^{c}+L_{FGR}\left(dB\right)$$

The standard form of the forest model in (2) is still used by the ITU Recommendation (ITU-R) model [17] for general forest environments in a wide frequency band (200 MHz–95 GHz). The A, B, and C parameters are then fitted to the free-space path loss model in (1), when the tree type and environment change to modify or compare with standard models.

### 2.2. Tree Attenuation Factors Model

**Figure 1:**
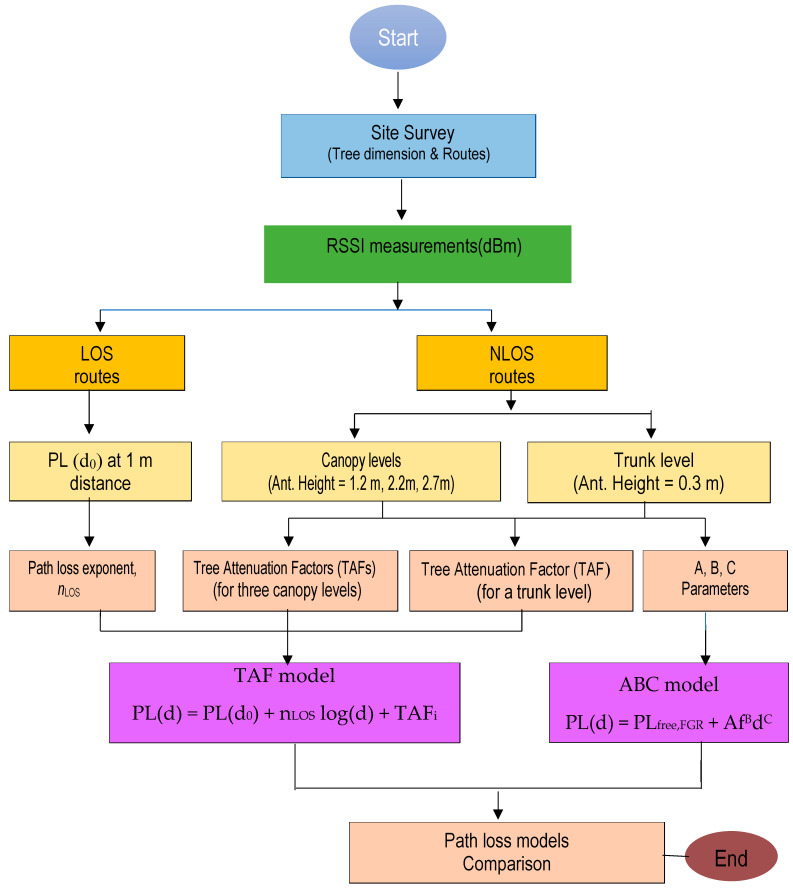
Flowchart of the proposed empirical path loss models.

The log-distance path loss models have usually been used to analyze indoor and outdoor electromagnetic wave propagation in the simple form as follows:

(7) $${PL}_{one\;slop}={PL(d}_{0})+10n{log}_{10}(d)$$

where ${PL(d}_{0})$ is path loss over the reference distance $d_{0}\left(1\;m\right),$ *d* is the distance between the transmitter and the receiver, and *n* is the path-loss exponent (PLE) that indicates how fast the path loss increases with distance. The empirical PLE is obtained as follows:

(8) $$n=[RSSI\left(d\right)-RSSI\left(d_{0}\right)]/10{log}_{10}(d)$$

where RSSI(*d*) is the RSSI at distance *d*, and $RSSI\left(d_{0}\right)$ is the RSSI at 1 m distance ($d_{0})$ in dBm. Since the trees in this study are separated, every 5 m distance acts as a floor attenuation factor (FAF) on wireless communication inside a building [29]. Therefore, we proposed to adapt the forest model with a tree attenuation factor (TAF) for the specific forest environment as follows:

(9) $${PL}_{forest}={PL(d}_{0})+10n{log}_{10}(d)+\sum_{i=1}^{M}{TAF}_{i}$$

where ${TAF}_{i}$ represents the attenuation in a forest caused by *M* trees, the subscript *i* represents the number of direct waves through the trees, and $n_{LOS}$ is the PLE of the LOS route. Four different TAFs were derived for antenna heights of 0.3, 1.2, 2.2, and 2.7 m representing the trunk, bottom canopy, middle canopy, and top canopy levels, respectively. The forest attenuation factors were also classified into either trunk attenuation factor or canopy attenuation factor. The proposed models were completed from field measurements in the next section as shown by a flowchart in Figure 1.

## 3. Field Measurements

### 3.1. Site Description

**Figure 2:**
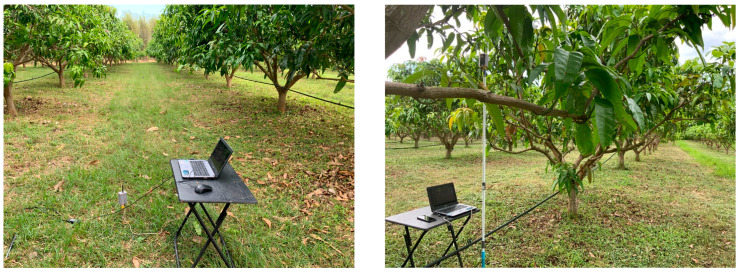
RSSI measurement for LOS (**left**) and NLOS (**right**).

**Figure 3:**
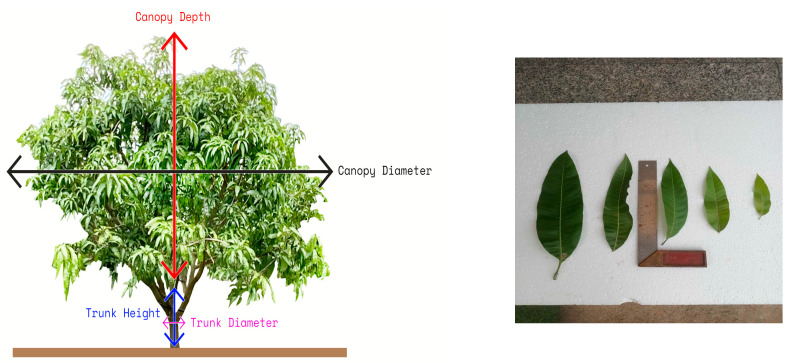
Parameters of a Ruby mango tree.

Measurements were taken at a Ruby mango plantation in Sakaeo Province of eastern Thailand (13.4166954° N, 102.1368925° E). To ensure a good harvest, Ruby mango trees must be planted at a certain density. Thus, the plantation follows a specific pattern. The trees were planted in straight lines with 6 m between rows and 5 m between trunks in the same row (Figure 2). There were 320 mango trees per hectare. Figure 3 (left) and Table 1 present the measured tree parameters. The trees had an average height of 4.5 m, which comprised the trunk height (0.55 m) and canopy depth (3.96 m), and an average canopy diameter of 5.69 m. Additionally, leaf dimensions were between 15–33 cm. as shown in Figure 3 (right).

### 3.2. Measurement Setup

**Figure 4:**
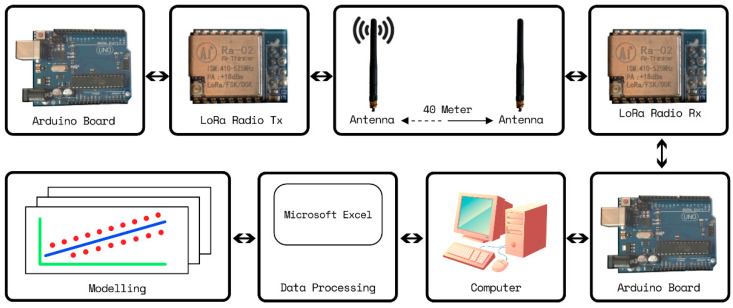
Measurement and modeling process block diagram.

**Figure 5:**
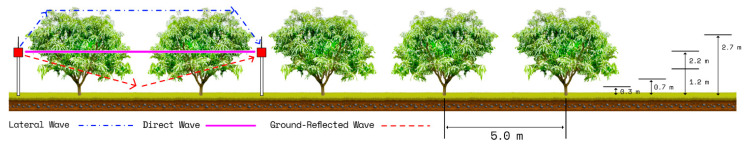
Propagation scenario.

The following equipment was used for propagation measurement: a fixed 433 MHz LoRa module (i.e., transceiver and omni-directional antenna) as the receiving station and a portable LoRa module as the transmitter. This frequency band is allowed only for the Asia region. This study used omni-directional antennas both as the transmitter and the receiver in order to obtain the surrounding effects in the proposed model. These modules were connected to a microcontroller (Arduino board) that programmed the transmitter to wirelessly send a data packet containing the word “hello” with the RSSI to the receiver every 1.5 s, as shown in Figure 4. Since this study was only focused on the wave propagation characteristics through the Ruby mango trees, therefore, a spreading factor (SF) of 7 and a bandwidth (BW) of 125 kHz were used to obtain the best RSSI and time on air. Table 2 summarizes the equipment parameters. To model the path loss, the RSSI data were captured by a notebook computer at the receiving station, while the portable transmitting node was moved in intervals of 5 m up to 40 m in both the forward and reverse directions. The antenna heights of the transmitter and receiver were varied at the same heights of 0.3, 1.2, 2.2, and 2.7 above the ground, as shown in Figure 5. This make waves propagate via the tree classified into three parts, namely (1) Ground-reflected wave, (2) Direct wave, and (3) Lateral wave. In order to obtain the correct measured data, the measurements were repeated three times, both forward and reverse routes without strong wind or rain. The measurements were divided to 2 routes for path loss modeling and validation with a total of 7680 measured RSSIs.

## 4. Results and Discussion

### 4.1. LOS Routes

**Figure 6:**
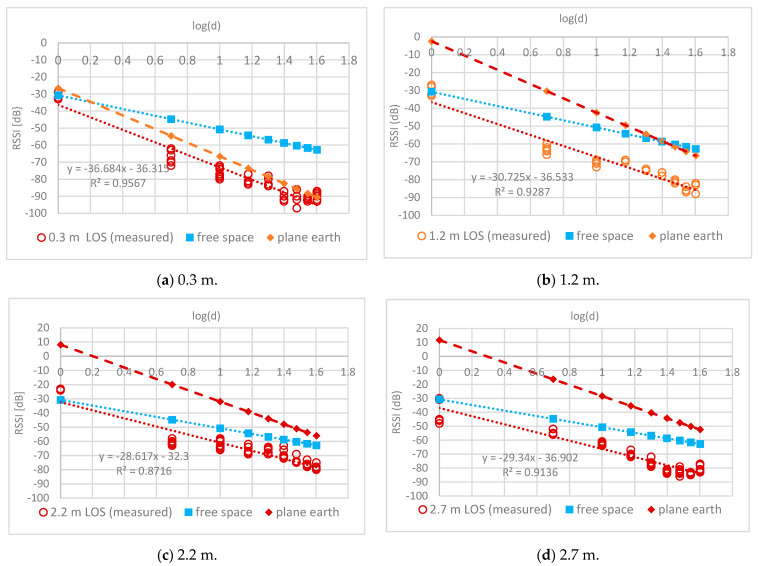
RSSI measurement (LOS) at different antenna heights.

To characterize the signal attenuation through trees, RSSI measurements were taken along LOS routes in a Ruby mango plantation. Data were measured along LOS routes every 5 m in a tree line for different antenna heights. Figure 6 shows the relationship between the RSSI and distance for one LOS route. The relationships at different antenna heights can be represented as follows:

- Trunk level (h = 0.3 m)

RSSI (d) = −36.32 − 36.68log(d) (10)

- Bottom canopy level (h = 1.2 m)

RSSI (d) = −36.5 − 30.7log(d)(11)

- Middle canopy level (h = 2.2 m)

RSSI (d) = −32.3 − 28.6log(d)(12)

- Top canopy level (h = 2.7 m)

RSSI (d) = −36.9 − 29.3log(d)(13)

At antenna heights close to the ground, the PLE was close to 4.0. In particular, (10) shows that the measured PLE agreed with the plane-Earth model in (3). The PLE decreased with increasing antenna height to approach 2.0. In particular, (12) and (13) show that the measured PLE agreed with the free-space model. Increasing the antenna height removed the fast fading due to strong winds in the cool season. The plane-Earth model was accurate when the antenna was near the ground, as shown in Figure 6a. As the antenna height increased, the plane-earth model introduced a large estimation error, especially at distances close to the receiving node as shown in Figure 6b,c.

### 4.2. NLOS Routes

**Figure 7:**
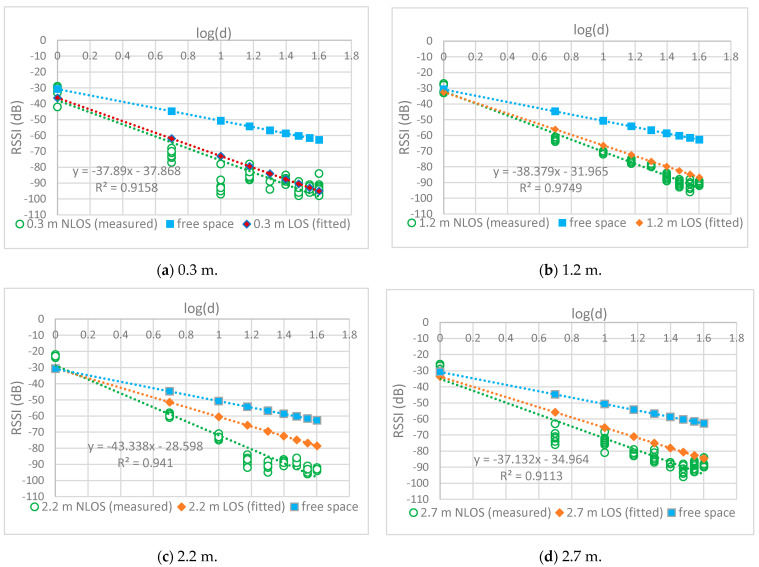
RSSI measurement (NLOS) at different antenna heights.

Figure 7 plots the relationship between the RSSI and distance when the effects of foliage loss were included at different antenna heights. These relationships are represented by the following equations:

- Trunk level (h = 0.3 m)

RSSI (d) = −37.87 − 37.89log(d)(14)

- Bottom canopy level (h = 1.2 m)

RSSI (d) = −31.97 − 38.38log(d)(15)

- Middle canopy level (h = 2.2 m)

RSSI (d) = −28.6 − 43.34log(d)(16)

- Top canopy level (h = 2.7 m)

RSSI (d) = −34.97 − 37.13log(d)(17)

The largest difference in RSSI between the LOS and NLOS routes was observed at an antenna height of 2.2 m, which corresponded to the largest PLE of 4.33. This is because radio waves traveling through the middle canopy suffered large attenuation. In contrast, a small difference was observed between the LOS and NLOS routes for waves propagating through the trunks, as shown in Figure 7a. This can be attributed to the near-ground effect, which includes diffraction at the trunk level. The difference between the LOS and NLOS routes was less at an antenna height of 1.2 m (Figure 7b) than at an antenna height of 2.2 m (Figure 7c). This is because waves propagating through the bottom canopy suffered less attenuation, owing to the less dense foliage. 

Similarly, the difference was also smaller at an antenna height of 2.7 m (Figure 7d) than at an antenna height of 2.2 m, because the waves propagated through less dense foliage in the top canopy, and the waves diffracted at the top of the canopy. The NLOS path loss model expressed by (14)–(17) was similar to the free-space model (CI).

**Figure 8:**
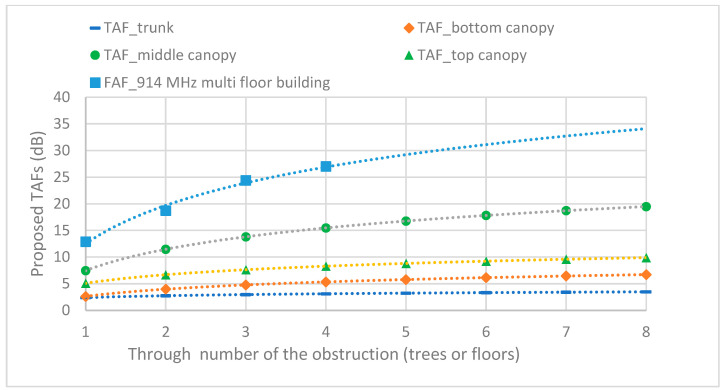
Proposed TAFs according to the number of obstructions (trees).

Table 3 presents the TAFs through the first to eighth trees. The middle canopy had the largest TAF because of the large canopy volume while the trunk had the lowest TAF because it presented the smallest obstruction. The validation of the proposed modes provided good agreements, especially in canopy levels because of the symmetry pattern. However, in the case of the trunk level, there was an error with RMSE for the validation since the trees in each row were not in a straight line. The table also presents the A, B, and C parameters in Equation (2). The B parameter was 0.39 for all cases which represent the single frequency of 433 MHz. Figure 8 shows that the proposed TAF followed an exponential curve according to the number of through trees, which is similar to through floors with FAF for multistory buildings [29]. From the above results, a suitable distance between the communication nodes is approximately 35–40 m with the specific SF and BW for network planning for this farm and similar environments.

### 4.3. Model Comparison

To compare the proposed model with the industry standard models in the literature, the RSSI needed to be converted into the path loss:

(18) $$PL\;\left(dB\right)=P_{t}+G_{t}+G_{r}-(RSSI+K)$$

where *K* is an offset that depends on the characteristics of the transceiver chip, the frequency, and the chosen technology. In the present study, LoRa communication was used with an SF of 7 and BW of 125 kHz [30].

The K value can be obtained by calibration. The following three conventional forest models were selected for comparison.

(1) ITU-R Foliage Attenuation Model

The ITU Recommendation (ITU-R) [31] was developed from UHF measurements and was proposed for cases where either the transmitting or receiving antenna is close to a small grove of trees, so that the majority of the signal propagates through the trees. ITU-R is commonly used for frequencies of 200 MHz–95 GHz and is expressed by

(19) $$ITU-R\;\left(dB\right)={0.2\;f}^{0.3}d^{0.6}$$

(2) COST 235 Model

The COST 235 model [32] is based on measurements made at millimeter-wave frequencies (9.6–57.6 GHz) through a small grove of trees over two seasons: in-leaf and out-of-leaf. This model is also applicable to frequencies of 200 MHz–95 GHz, and is expressed by

(20) $$COST\;235\;\left(dB\right)=\left\{\begin{array}{c} {26.6\;f}^{-0.2}d^{0.5}\;\;\;\;\;\;\;\mathrm{out}\;\mathrm{of}\;\mathrm{leaf}\\ {15.6\;f}^{-0.009}d^{0.26}\;\;\;\;\;\;\;\;\;\;\mathrm{in}\;\mathrm{leaf} \end{array}\right.$$

For both ITU-R and COST 235, *f* is the frequency (MHz), and *d* is the tree depth (m).

(3) FITU-R Foliage Attenuation with Plane-Earth Model

FITU-R was developed by considering datasets collected during two foliation states at 11.2 and 20 GHz [33]:

(21) $$FITU-R\;\left(dB\right)=\left\{\begin{array}{c} 0.37f^{0.18}d^{0.59\;}\;\;out\;of\;leaf\\ 0.39f^{0.39}d^{0.25}\;\;\;\;\;\;\;\;\;\;\;in\;leaf \end{array}\right.\;$$

Lateral waves become dominant at relatively large tree depths, especially in the VHF and UHF bands when both the transmitter and receiver are placed inside the forest. Based on measurement data taken from an oil palm plantation, the model becomes [18]:

(22) $$\;LITU\left(dB\right)={0.48\;f}^{0.43}d^{0.13}+40\log\left(d\right)-20\log\left(h_{t}\right)-20\log(h_{r})$$

where *f* is the carrier frequency (MHz), *h_t_* is the transmitting antenna height (m), *h_r_* is the receiving antenna height (m), and *d* is the distance between the transmitter and receiver (m).

To observe the deviation of the measurement and related empirical models, RMSE (root-mean-square-error) and MAE (mean-absolute-error) were calculated as follows:

(23) $$RMSE=\sqrt{\frac{\sum_{i=1}^{N}{\left({MEA}_{i}-{PL}_{i}\right)}^{2}}{N}}$$

and

(24) $$MAE=\frac{\sum_{i=1}^{N}M_{i}-{PL}_{i}}{N}$$

where $M_{i}$ is measured path loss, ${PL}_{i}$ is predicted path loss, *N* is a total number of the data, and subscripts *i* are the number of the data.

Table 3 indicates that the maximum TAF through eight trees (19.48 dB) was obtained for the middle canopy level, and the minimum TAF (3.49 dB) was obtained at the trunk level. The MAEs of the models are compared in Table 4.

**Figure 9:**
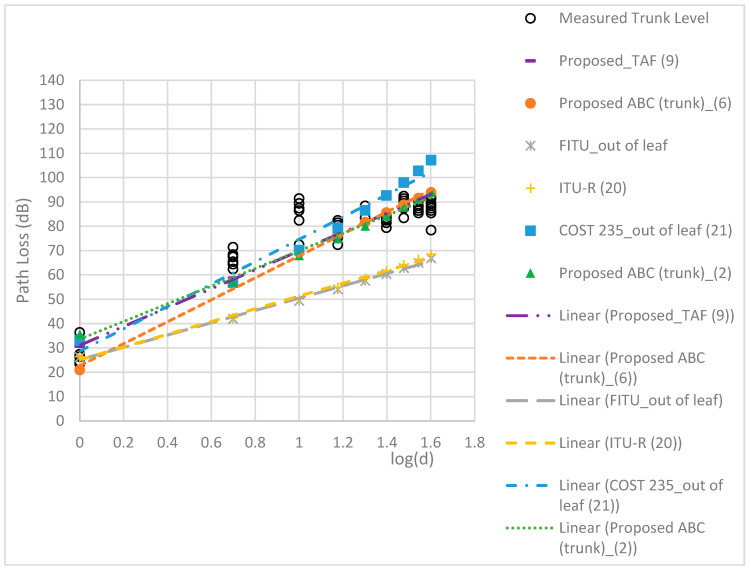
Comparison of models at the trunk level.

**Figure 10:**
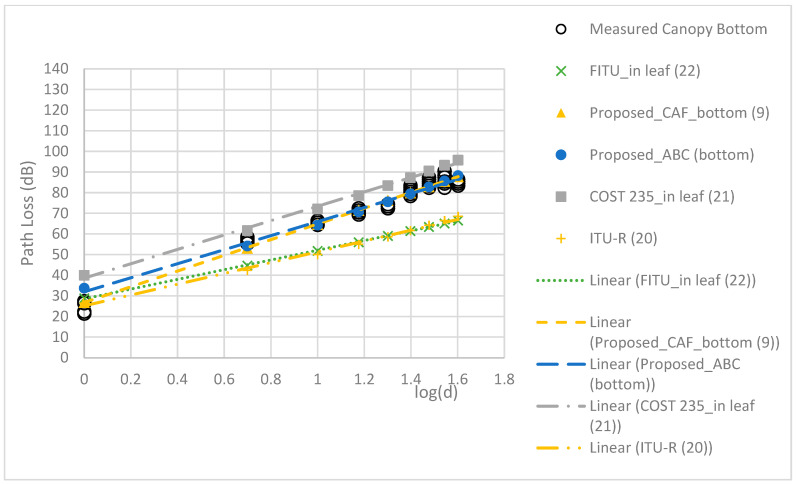
Comparison of models at the bottom canopy level.

**Figure 11:**
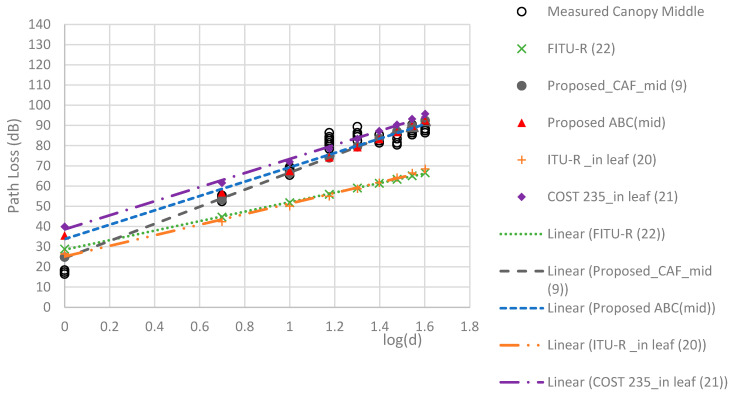
Comparison of models at the middle canopy level.

**Figure 12:**
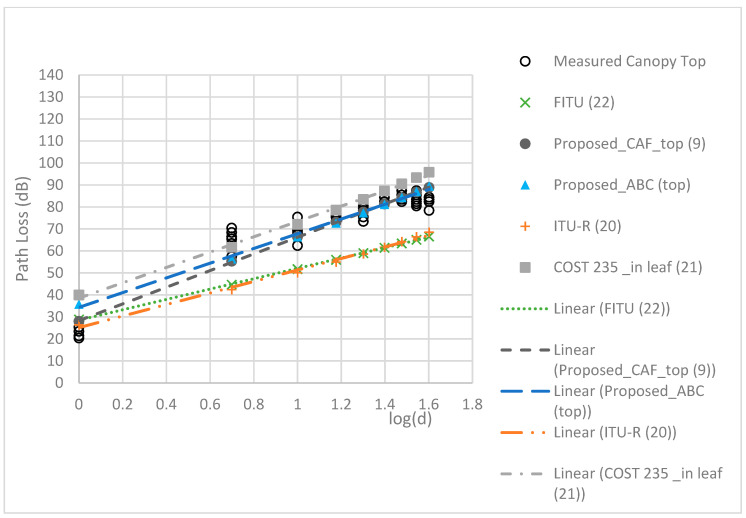
Comparison of models at the top canopy level.

The RMSEs of the models are compared in Table 5. The proposed TAF models provided a more accurate prediction than the three conventional models, with the best MAE of 2.22 dB and RMSE of 2.65 dB. Figure 9, Figure 10, Figure 11 and Figure 12 compare the proposed models with the three conventional models at different antenna heights. Note that the large deviation of measured path loss occurred at the trunk level with a MAE of 4.79 and RMSE of 6.2 (see Figure 9). Additionally, the MAE was smaller than the RMSE because of the deviation of the path loss measurement at the bottom canopy. (see Figure 10). The models provide minimum path loss since there are a little leaf in the bottom canopy level. The proposed ABC model provided a good prediction accuracy overall, however the proposed TAF model generally provided the best prediction accuracy because of influence from TAFs through the trees. At the trunk level, the out-of-leaf ITU-R and FITU-R models had a large error, with RMSE of 21.65 dB and 22.59 dB, respectively, while the out-of-leaf COST 235 model demonstrated a better prediction accuracy with RMSE of 11.77 dB, as shown in Table 5. At the canopy levels, the proposed TAF model still demonstrated good prediction accuracy, especially at the bottom canopy level, which had the lowest leaf density. Similarly, the proposed ABC model still provided good prediction accuracy at distances greater than 10 m. The in-leaf COST 235 model also performed better than the in-leaf ITU-R and FITU models.

## 5. Conclusions

In this study, an empirical path loss model was proposed for a 433 MHz LPWAN in a symmetric vegetation plantation. RSSI measurements were taken in a Ruby mango plantation in Sakaeo, Thailand. LOS and NLOS propagation measurement data were collected to model the path loss at the trunk and three canopy levels. The PLEs at the trunk, bottom canopy, middle canopy, and top canopy levels were 3.67, 3.07, 2.86, and 2.93, respectively, for LOS propagation, and 3.79, 3,84, 4.33, and 3.71, respectively, for NLOS propagation. TAFs were obtained for up to eight trees. When compared with three conventional models (COST 235, ITU-R and FITU), the proposed TAF model provided the best prediction accuracy with an average MAE of 3.84 and RMSE of 4.93, while the proposed ABC model also performed well, with an average MAE of 4.58 and RMSE of 5.9. Therefore, the proposed TAF model is suitable for a symmetric pattern of Ruby mango trees in normal situations. However, when the tree produces flowers and fruit, the proposed models may provide an error for prediction. To reduce the MAE and RMSE, future work will involve the incorporation of artificial intelligence models, such as neural networks and adaptive neuro-fuzzy inference systems.

## Figures and Tables

**Table 1:** Measurement Parameters of Ruby Mango Trees (in Meters).

No.	Total Height	Trunk Height	Trunk Diameter	Canopy Depth	Canopy Diameter
Tree 1	3.82	0.56	0.40	3.4	5.5
Tree 2	4.66	0.66	0.56	4.0	6.0
Tree 3	4.79	0.49	0.45	4.3	5.6
Tree 4	5.15	0.65	0.64	4.5	6.5
Tree 5	4.77	0.47	0.63	4.3	6.2
Tree 6	3.96	0.46	0.46	3.5	4.7
Tree 7	4.85	0.65	0.54	4.2	6.0
Tree 8	3.97	0.47	0.43	3.5	5.0
**Average**	**4.50**	**0.55**	**0.51**	**3.96**	**5.69**

**Table 2:** Measurement Setup.

No.	Parameters	Value	Unit
1	Power Amplifier (PA)	18	dBm
2	Antenna gain	2.2	dBi
3	Frequency	433	MHz
4	Bandwidth (BW)	125	kHz
5	Spreading factor	7	-
6	Code rate (CR)	4/5	-
7	Antenna height	0.3–2.7	m

**Table 3:** TAFs and ABC Parameters at 433 MHz (SF = 7, BW = 125 KHz).

Antenna Height (m)	PLE (LOS)	PLE (NLOS)	Tree Attenuation Factors		A	B	C	Validation (RMSE)
			Through	TAF (dB)				
0.3 (trunk)	3.67	3.79	1	2.40	0.98	0.39	0.34	2.11
			2	2.76				
			3	2.97				
			4	3.12				
			5	3.24				
			6	3.34				
			7	3.42				
			8	3.49				
1.2 (bottom canopy)	3.07	3.84	1	2.62	0.8	0.39	0.35	0.42
			2	3.98				
			3	4.79				
			4	5.35				
			5	5.79				
			6	6.15				
			7	6.46				
			8	6.72				
2.2 (middle canopy)	2.86	4.33	1	7.46	0.98	0.39	0.33	0.31
			2	11.47				
			3	13.81				
			4	15.47				
			5	16.76				
			6	17.82				
			7	18.71				
			8	19.48				
2.7 (top canopy)	2.93	3.71	1	5.09	1.0	0.39	0.3	1.18
			2	6.70				
			3	7.63				
			4	8.30				
			5	8.82				
			6	9.24				
			7	9.60				
			8	9.91				

**Table 4:** Mean Absolute Error Comparison of Models.

Antenna Height (m)	MAE (dB)				
	Proposed		ITU-R	COST235	FITU-R
	TAF	ABC			
0.3 (trunk)	4.79	5.54	19.63	10.19	20.55
1.2 (bottom canopy)	2.22	2.66	16.39	7.91	16.49
2.2 (middle canopy)	4.21	5.14	19.08	6.86	19.09
2.7 (top canopy)	4.13	4.96	17.69	7.45	17.84
Average	3.84	4.58	18.2	8.1	18.49

**Table 5:** Root Mean Square Error Comparison of Models.

Antenna Height (m)	RMSE (dB)				
	Proposed		ITU-R	COST235	FITU-R
	TAF	ABC			
0.3 (trunk)	6.2	7.08	21.65	11.77	22.59
1.2 (bottom canopy)	2.65	3.69	16.96	8.61	17.10
2.2 (middle canopy)	5.61	6.70	19.85	8.63	19.10
2.7 (top canopy)	5.26	6.12	18.62	9.09	18.53
Average	4.93	5.90	19.27	9.53	19.33

## Data Availability

Data are contained within the article.
